# Improved adequacy of levothyroxine treatment in low-risk differentiated thyroid cancer using the 2025 ATA criteria: a multicenter real-world analysis of 1,016 patients

**DOI:** 10.1530/ETJ-26-0066

**Published:** 2026-06-30

**Authors:** Juan J Díez, Emma Anda, Victoria Alcazar, Juan C Galofré, Cristina Familiar, María J Pamplona-Civera, Silvia González-Martínez, Ana Herrero-Ruiz, María R Alhambra, Alejandra Planas, Cecilia Sánchez-Ragnarsson, Tomás Martín-Hernández, Beatriz Rodriguez-Jiménez, Mireia Mora, Aida Orois, Reinaldo Sánchez-Barrera, Julia Sastre

**Affiliations:** ^1^ Department of Endocrinology, Hospital Universitario Puerta de Hierro Majadahonda, Majadahonda, Spain; ^2^ Instituto de Investigación Sanitaria Puerta de Hierro Segovia de Arana, Majadahonda, Spain; ^3^ Department of Medicine, Universidad Autónoma de Madrid, Madrid, Spain; ^4^ Department of Endocrinology, Hospital Universitario de Navarra, Pamplona, Spain; ^5^ Department of Endocrinology, Hospital Severo Ochoa, Leganés, Spain; ^6^ Department of Endocrinology, Clínica Universidad de Navarra, Pamplona, Spain; ^7^ Department of Endocrinology, Hospital Clínico San Carlos, Madrid, Spain; ^8^ Department of Endocrinology, Hospital Royo Villanova, Zaragoza, Spain; ^9^ Department of Endocrinology, Hospital Universitario de Cabueñes, Gijón, Spain; ^10^ Instituto de Investigación Sanitaria del Principado de Asturias (ISPA) Oviedo, Oviedo, Spain; ^11^ Department of Endocrinology, Hospital Universitario de Salamanca, Salamanca, Spain; ^12^ UGC Endocrinología y Nutrición, Hospital Universitario Reina Sofía, Córdoba, Spain; ^13^ Instituto Maimónides de Investigación Biomédica de Córdoba (IMIBIC), Córdoba, Spain; ^14^ Department of Endocrinology, Vall d’Hebron Hospital Campus, Barcelona, Spain; ^15^ Department of Endocrinology, Hospital Universitario Central de Asturias, Oviedo, Spain; ^16^ Department of Endocrinology, Hospital Universitario Virgen Macarena, Sevilla, Spain; ^17^ Department of Endocrinology, Hospital Clínic, Barcelona, Spain; ^18^ Department of Endocrinology, Hospital Universitario de Bellvitge, Barcelona, Spain; ^19^ Department of Endocrinology, Hospital Universitario de Toledo, Toledo, Spain

**Keywords:** differentiated thyroid cancer, treatment adequacy, levothyroxine therapy, thyrotropin suppression, dynamic risk stratification, ATA guidelines

## Abstract

**Background:**

Levothyroxine titration is essential in low-risk differentiated thyroid cancer (DTC).

**Objective:**

To compare levothyroxine treatment adequacy, response distribution, and determinants of inadequacy in a large real-world cohort applying the 2015 versus 2025 American Thyroid Association (ATA) criteria.

**Methods:**

We conducted a multicenter, retrospective cohort study including 1,016 adults with low-risk DTC (median follow-up: 79 months). Dynamic risk stratification and levothyroxine treatment adequacy – defined according to framework-specific thyrotropin (TSH) targets – were assessed at 12 months and at the last visit. Secondary outcomes included rates of overtreatment and undertreatment. Multivariable logistic regression identified factors associated with adequacy among excellent or indeterminate responders.

**Results:**

Under the ATA-2025 criteria, the proportion of patients with an excellent response increased to 72.0% at 12 months and 86.9% at last visit, compared with 62.3 and 77.8% using the ATA-2015 criteria. Treatment adequacy changed from 26.0% at 12 months and 39.6% at the last follow-up visit with ATA-2015 to 40.7 and 67.4% with ATA-2025 (both *P* < 0.001). Under ATA-2015, inadequacy was driven by overtreatment among excellent responders (61.8% at 12 months; 30.5% at final evaluation) and undertreatment among indeterminate responders (48.4%; 62.2%). Applying the ATA-2025 criteria reclassified overtreated excellent responders as 51.9 and 25.5% and undertreated indeterminate responders as 6.6 and 8.8%, respectively. At the last visit, older age modestly favored adequacy (OR: 1.02 per year, 1.00–1.03) and levothyroxine dose instability strongly predicted inadequacy (OR: 0.12 for adequacy, 0.08–0.16).

**Conclusion:**

Applying the ATA-2025 criteria increases the proportion of patients classified as having adequate TSH control and decreases the proportion labeled as over- or undertreated under the 2015 framework, reflecting the broader, risk-adapted TSH targets introduced in 2025.

## Introduction

Differentiated thyroid cancer (DTC) is the most frequent endocrine malignancy, with incidence increasing over recent decades (1), yet most patients still present with low-risk tumors and near-normal survival rates approaching 100% (2, 3). After initial treatment, levothyroxine therapy is required both to maintain euthyroidism and to modulate TSH according to recurrence risk and therapeutic response (4, 5). Appropriate titration is crucial to prevent underdosage, which may favor recurrence, and to avoid excessive suppression, linked to cardiovascular and skeletal complications (4, 6). Compared with the 2015 guidelines (7), the 2025 American Thyroid Association (ATA) update (8) introduces major changes to initial risk assessment, dynamic risk stratification (DRS), and criteria for levothyroxine adequacy. The recurrence risk system now includes four categories (low, low-intermediate, intermediate-high, and high), enabling more individualized classification, while the low-risk group remains largely unchanged. The updated DRS also provides clearer definitions of the four response categories – excellent, indeterminate, biochemically incomplete, and structurally incomplete – based on biochemical and imaging findings (8).

The criteria for assessing levothyroxine therapy adequacy have been updated, particularly regarding TSH targets for each response category. TSH goals are now more individualized and generally less restrictive for patients with favorable responses and low recurrence risk. Those with an excellent or indeterminate response may maintain TSH within the reference range, whereas patients with biochemical or structural incomplete responses are advised to keep TSH below the normal interval (8).

Under the 2015 criteria, many patients who were disease-free (excellent response) or had minimal residual disease (indeterminate response) were overtreated, exposing them to unnecessary risks from excessive TSH suppression. In our prior cohort of 1,016 low-risk patients, adequacy was only 26.0% at 12 months and 39.5% at the final follow-up (9). We hypothesize that, although patients with an excellent response and TSH < 0.5 mIU/mL would remain classified as overtreated, some previously considered undertreated (TSH > 2 mIU/mL) may now meet adequacy under the 2025 criteria. Likewise, among indeterminate responders, certain patients formerly labeled undertreated (TSH: > 0.5 mIU/mL) would now be adequately controlled, whereas some previously adequate cases (TSH: 0.1–0.5 mIU/mL) may be reclassified as overtreated. On this basis, the present study evaluates treatment adequacy in the previously analyzed multicenter Spanish cohort using the updated ATA-2025 TSH targets.

## Methods

### Subjects and study design

This multicenter, retrospective study is described in detail in our previous report (9). In summary, eligible patients were older than 18 years and had a histological diagnosis of low-risk DTC. Initial risk classification was performed according to the ATA-2015 risk-of-recurrence system, which was the prevailing guideline during the entire recruitment period. Although the ATA-2025 guidelines introduce refined categories (low, low-intermediate, intermediate-high, and high) with histology-specific nuances – particularly for papillary thyroid carcinoma (PTC) multifocality and for follicular thyroid carcinoma/invasive encapsulated follicular variant of papillary thyroid carcinoma (FTC/IEFVPTC), and oncocytic thyroid carcinoma (OTC) – we verified that all multifocal tumors in this cohort were intrathyroidal, without aggressive histology, vascular invasion, or clinically evident nodal disease. No FTC, IEFVPTC, or OTC cases fulfilled the ATA-2025 criteria for low-intermediate or intermediate-high risk.

Treatments employed in this cohort included lobectomy or total/near-total thyroidectomy, with or without subsequent radioiodine (RAI) ablation. The widespread use of radioiodine in this patient cohort reflects the real-world practice before 2025, a period in which RAI was commonly administered to low-risk patients following total thyroidectomy according to institutional protocols and prevailing interpretations of the 2009 and 2015 ATA guidelines. These guidelines allowed selective use of adjuvant RAI in low-risk DTC, contributing to broad utilization across participating centers. Clinical, pathological, and biochemical data, as well as imaging findings during follow-up, were collected, together with the information required for the DRS at 12 months after initial treatment and at the last available follow-up visit within the study period (January 1 to December 31, 2024). The presence of hypoparathyroidism, comorbidities potentially affecting levothyroxine dosing, and – among patients with more than two years of follow-up – the stability or instability of levothyroxine dosage were also recorded. Patients whose levothyroxine dose remained unchanged over the past year were classified stable, whereas those who had undergone one or more dose adjustments during that period were considered unstable. For patient classification at diagnosis, the tumor–node–metastasis (TNM) staging system was applied according to the criteria of the American Joint Committee on Cancer (AJCC/UICC), 8th edition (10).

All patients’ data were obtained under the standard medical care conditions. This study was conducted in accordance with the Declaration of Helsinki (as revised in 2013). The patient’s confidential information was protected according to national law, and the study was approved by the Local Ethics Committee of the Hospital Universitario Puerta de Hierro Majadahonda (Madrid, Spain) (PI 157/24).

### Laboratory procedures

Serum thyroglobulin (Tg) and Tg antibodies (TgAbs) were measured using the standard procedures implemented at each participating hospital. The instruments used for Tg quantification, together with the corresponding number of patients, were as follows: Alinity (Abbott, USA; 84 patients), DXI (Beckman, USA; 139 patients), Liaison (Diasorin, Italy; 40 patients), Cobas (Roche, Switzerland; 346 patients), Elecsys (Roche, 153 patients), Atellica (Siemens, Germany; 172 patients), and Immulite 2000 (Siemens, 82 patients). The limit of detection for all Tg assays was <0.2 ng/mL, except for Immulite 2000, which had a detection limit of 0.5 ng/mL. TgAbs were interpreted qualitatively (positive/negative) according to the manufacturer-specific reference ranges used in each participating laboratory. Because several different analytical platforms were employed across centers, a unified numerical cutoff was not applied. This qualitative classification was used consistently for DRS.

Table S1 (Supplementary Material (see section on Supplementary materials given at the end of the article)) summarizes the methods used by investigators to measure serum TSH and the corresponding reference intervals for each instrument. The lower limit of the TSH reference interval ranged from 0.25 to 0.55 μIU/mL, and the upper limit ranged from 4.2 to 5.3 μIU/mL. All participating clinical laboratories employed standardized assays and complied with established quality standards.

### Dynamic risk stratification

For the assessment of DRS, we considered biochemical data (Tg and TgAbs) and imaging studies (ultrasound and additional modalities when required), as well as the type of treatment received by each patient (thyroidectomy with RAI ablation, thyroidectomy without ablation, or lobectomy). Tg measurements were considered valid only when TgAb titers were negative.

According to the ATA-2025 criteria, patients undergoing total thyroidectomy with RAI ablation were classified as having an excellent response when non-stimulated Tg was <0.2 ng/mL (<0.50 ng/mL with Immulite 2000) or stimulated Tg was < 1 ng/mL, with negative imaging. Indeterminate response included non-specific imaging findings, non-stimulated Tg 0.2–1 ng/mL (0.5–1 ng/mL for Immulite 2000), stimulated Tg 1–10 ng/mL, or stable/declining TgAbs. A biochemical incomplete response was defined by non-stimulated Tg > 1 ng/mL, stimulated Tg > 10 ng/mL, or rising TgAbs, in the absence of structural disease. In patients treated with total thyroidectomy without RAI, an excellent response corresponded to non-stimulated Tg < 2.5 ng/mL; an indeterminate response corresponded to non-specific imaging, Tg 2.5–5 ng/mL, or stable/declining TgAbs; and a biochemical incomplete response corresponded to Tg > 5 ng/mL or increasing TgAbs with negative imaging. After lobectomy, an excellent response required normal or low-risk contralateral nodules, benign cytology, and no abnormal lymph nodes; indeterminate and biochemical incomplete categories were not applied. Across all groups, structural incomplete response was assigned when imaging or biopsy confirmed local or distant disease.

### Adequacy of levothyroxine treatment

The adequacy of levothyroxine therapy was evaluated based on the serum TSH concentration at 12 months after initial treatment and at the last follow-up visit. In patients who achieved an excellent or indeterminate response, treatment was considered adequate when the serum TSH level was within the reference range established by each participating hospital’s local laboratory. In cases of biochemical or structural incomplete response, a TSH level below the local reference range was deemed appropriate. In all cases, TSH concentrations above the target range indicated insufficient levothyroxine dosing, whereas levels below the range were indicative of overtreatment.

### Statistical analysis

Quantitative variables are expressed as median (interquartile range (IQR)). Categorical variables are described using absolute values, ratios, or percentages. Proportions were compared using the chi-square test or Fisher’s exact test. Comparisons of quantitative variables between two groups were conducted using the Mann–Whitney U test. The McNemar test was used to compare treatment adequacy at 12 months and the last follow-up visit (paired data). Several univariate and multivariable logistic regression models were applied to identify factors influencing levothyroxine therapy adequacy at 12 months and at the last follow-up visit. We used two multivariable logistic regression models. The first included only demographic features (gender and age), while the second included the former plus clinical, pathological, and therapeutic variables related to the prognosis of DTC. Chronic hypoparathyroidism was included in the model as a clinically relevant postoperative variable with potential influence on levothyroxine absorption through calcium or calcitriol supplementation. Its inclusion was exploratory, and it was not hypothesized *a priori* to be a determinant of treatment adequacy. All statistical tests were two-sided, and differences were considered significant when *P* < 0.05.

## Results

### Included patients

A total of 1,016 patients had available data for evaluation at 12 months, and 978 had sufficient data for assessment at the last follow-up visit (Table 1). In both cases, most patients were women with stage I papillary thyroid carcinoma who had undergone total thyroidectomy. Approximately two-thirds of the patients had received RAI ablation. The median age of the cohort was 48 years, and the median tumor size was 1.2 cm. The median follow-up duration was 79 (42–142) months. Of the 1,016 included subjects, levothyroxine dose stability was evaluated in 898 (88.4%), as their follow-up exceeded two years. Dose instability was identified in 310 of these patients (34.5%). Among the 81 patients treated with lobectomy alone, 52 (64.2%) were receiving levothyroxine at 12 months, whereas 29 (35.8%) remained euthyroid without replacement therapy. Dose stability was evaluated only in patients on levothyroxine; individuals not receiving LT4 were not classified as ‘stable’ by definition, as dose stability does not apply in the absence of replacement therapy.

**Table 1:** Main clinical characteristics of the studied patients. Data are presented as median (IQR) for quantitative variables and as *n* (%) for categorical variables.

	Patients evaluated at	
	12 months	Last visit
*n*	1,016	978
Gender		
Male	196 (19.3)	187 (19.1)
Female	820 (80.7)	791 (80.9)
Age, years	48.0 (38.0–59.0)	48.0 (38.0–59.0)
Histology		
Papillary	929 (91.4)	895 (91.5)
Follicular	87 (8.6)	83 (8.5)
Tumor size, cm	1.2 (0.7–2.0)	1.2 (0.7–2.0)
Multifocality	372 (36.6)	360 (36.8)
Chronic lymphocytic thyroiditis	291 (28.6)	282 (28.8)
Incidental	242 (23.8)	238 (24.3)
Surgery		
Lobectomy	81 (8.0)	74 (7.6)
Total thyroidectomy	935 (92.0)	904 (92.4)
Radioiodine	667 (65.6)	648 (66.3)
TNM stage		
I	984 (96.9)	949 (97.0)
II	32 (3.1)	29 (3.0)

### Dynamic risk stratification

Using the ATA-2025 criteria, at 12 months after treatment, 72.0% of patients showed an excellent response and 25.4% an indeterminate response. The proportion of patients with biochemical incomplete response (1.8%) and structural incomplete response (0.8%) was minimal. This distribution of treatment response at 12 months differed significantly (*P* < 0.001) from that obtained with the 2015 criteria, due to the increase in the percentage of patients classified as having an excellent response (Table 2).

**Table 2:** Dynamic risk stratification and adequacy of levothyroxine therapy at 12 months and at the last visit according to the ATA-2015 and ATA-2025 criteria. Data are presented as *n* (%) for categorical variables.

	12 months (*n* = 1,016)			Last visit (*n* = 978)		
	ATA-2015	ATA-2025	*P*	ATA-2015	ATA-2025	*P*
Dynamic risk stratification			<0.001			<0.001
Excellent	633 (62.3)	732 (72.0)		761 (77.8)	850 (86.9)	
Indeterminate	345 (34.0)	258 (25.4)		193 (19.7)	113 (11.6)	
Biochemical incomplete	30 (3.0)	18 (1.8)		20 (2.0)	11 (1.1)	
Structural incomplete	8 (0.8)	8 (0.8)		4 (0.4)	4 (0.4)	
Adequacy of levothyroxine therapy			<0.001			<0.001
Adequate	264 (26.0)	414 (40.7)		387 (39.6)	659 (67.4)	
Excess	485 (47.7)	537 (52.9)		266 (27.2)	255 (26.1)	
Insufficient	267 (26.3)	65 (6.4)		325 (33.2)	64 (6.5)	

**Figure 1:**
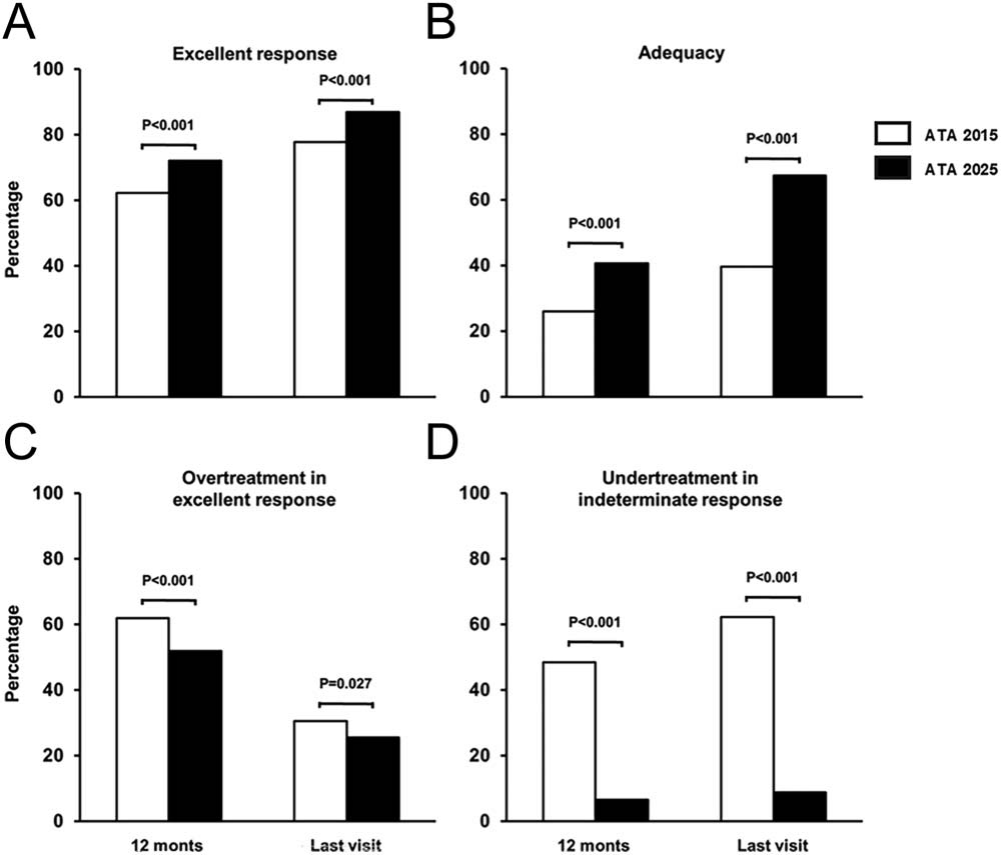
Percentage of patients with an excellent response to treatment (A), adequacy of levothyroxine therapy (B), overtreatment among patients with an excellent response (C), and undertreatment among patients with an indeterminate response (D). Each panel displays the percentage of patients evaluated at 12 months and at the last follow-up visit. The white bars correspond to evaluation using the ATA-2015 criteria, whereas the black bars correspond to evaluation using the ATA-2025 criteria.

At the last follow-up visit, the proportion of patients with an excellent response had increased compared with the 12-month assessment, reaching 86.9% (850/978; *P* < 0.001; McNemar test). Correspondingly, the proportions of indeterminate, biochemical incomplete, and structural incomplete responses decreased to 11.6, 1.1, and 0.4%, respectively. Again, comparison with the ATA-2015 criteria showed statistically significant differences (*P* < 0.001), attributable to the higher percentage of excellent responses when applying the ATA-2025 criteria at the last visit (Fig. 1A).

### Adequacy at 12 months and at last visit

Using the ATA-2025 criteria, adequacy of levothyroxine treatment was 40.7% at 12 months and increased significantly to 67.4% (659/978; *P* < 0.001; McNemar test) at the last visit (Fig. 1B). At both time points, this proportion was significantly higher than that obtained using the 2015 criteria (26.0 and 39.6%, respectively; *P* < 0.001). This improvement in the proportion of patients classified as adequately treated was primarily attributable to the reduction in the proportion of patients with insufficient levothyroxine dosing, both at 12 months (6.4 vs 26.3% with the 2015 criteria) and at the last visit (6.6 vs 33.2% with the 2015 criteria) (Table 2).

Table 3 shows the percentages of treatment adequacy according to DRS when applying the two ATA criteria. In all cases, the proportion of patients adequately controlled was higher (Fig. 1B), and the proportions of overtreatment and undertreatment were lower (Fig. 1C and D), with the 2025 criteria than with the 2015 criteria, both at 12 months and at the last visit.

**Table 3:** Number and proportion of patients with adequate and inadequate control at 12 months and at the last visit classified according to dynamic risk stratification using the ATA-2015 and ATA-2025 criteria. Data are presented as *n* (%) for categorical variables.

	ATA-2015				ATA-2025			
	Excellent	Indeterminate	Biochemical incomplete	Structural incomplete	Excellent	Indeterminate	Biochemical incomplete	Structural incomplete
Adequacy at 12 months (*n* = 1,016)								
Number	633	345	30	8	732	258	18	8
Adequate	163 (25.8)	94 (27.2)	4 (13.1)	3 (37.5)	313 (42.8)	85 (32.9)	12 (66.7)	4 (50.0)
Excess	391 (61.8)	84 (24.3)	10 (33.3)	0	380 (51.9)	156 (60.5)	1 (5.6)	0
Insufficient	79 (12.5)	167 (48.4)	16 (53.3)	5 (62.5)	39 (5.3)	17 (6.6)	5 (27.8)	4 (50.0)
Adequacy at last visit (*n* = 978)								
Number	761	193	20	4	850	113	11	4
Adequate	337 (44.3)	46 (23.8)	3 (15.0)	1 (25.0)	585 (68.8)	65 (57.5)	7 (63.6)	2 (50.0)
Excess	232 (30.5)	27 (14.0)	7 (35.0)	0	217 (25.5)	38 (33.6)	0	0
Insufficient	192 (25.2)	120 (62.2)	10 (50.0)	3 (75.0)	48 (5.6)	10 (8.8)	4 (36.4)	2 (50.0)

For comparative analysis, two groups of patients were examined: those with an excellent or indeterminate response, whose TSH target according to the ATA-2025 criteria is to maintain levels within the reference range, and those with an incomplete biochemical or structural response, for whom a TSH target below the reference range is recommended. In patients with an excellent or indeterminate response, the higher proportion of inadequacy with the ATA-2025 criteria was attributable to excessive levothyroxine dosing, both at 12 months (51.9 and 60.5%, respectively) and at the last visit (25.5 and 33.6%, respectively). In the small group of patients with a biochemical or structural incomplete response, inadequacy was mainly due to insufficient dosing (Table 3).

### Comparison of adequately and inadequately controlled patients

We compared patients with adequate and inadequate control within the group of subjects with an excellent or indeterminate response at 12 months (*n* = 990). Patients with adequate control (TSH within the normal range) were more likely to be male and have papillary histology, smaller tumor size, unifocal tumors, incidental tumors, treatment with lobectomy, and no treatment with RAI (Table 4). When adequacy was assessed at the last follow-up visit (*n* = 963), patients with adequate control were older, had a longer follow-up duration, had a higher proportion of lobectomy, and had a lower proportion of dose instability. When comparing patients with adequate control (TSH below the normal range) and inadequate control in the groups with an incomplete biochemical or structural response, no significant differences were found either at 12 months (*n* = 26) or at the last visit (*n* = 15) (Table S2, Supplementary Material).

**Table 4:** Comparison of patients with adequacy and inadequacy of levothyroxine treatment in the group of subjects with excellent or indeterminate response at 12 months and at the last follow-up visit according to the ATA-2025 criteria (TSH within the normal reference range). Data are presented as median (IQR) for quantitative variables and as *n* (%) for categorical variables.

	Adequacy of levothyroxine therapy in patients with excellent or indeterminate response					
	Evaluation at 12 months (*n* = 990)			Evaluation at the last visit (*n* = 963)		
	Adequate	Inadequate	*P* value	Adequate	Inadequate	*P* value
*n*	398	592		650	313	
Gender			<0.001			0.486
Male	103 (25.9)	88 (14.9)		129 (19.8)	56 (17.9)	
Female	295 (74.1)	504 (85.1)		521 (80.2)	257 (82.1)	
Age, years	49.0 (39.8–59.0)	47.5 (38.0–59.0)	0.267	49.0 (39.0–59.0)	46.0 (36.5–58.5)	0.043
Time of follow-up, months	—	—		87.5 (52.0–153.0)	74.0 (39.5–124.5)	0.001
Histology			0.037			0.261
Papillary	373 (93.7)	532 (89.9)		592 (91.1)	292 (93.3)	
Follicular	25 (6.3)	60 (10.1)		58 (8.9)	21 (6.7)	
Tumor size, cm	1.0 (0.5–1.7)	1.4 (0.8–2.2)	<0.001	1.2 (0.7–2.0)	1.3 (0.7–2.0)	0.291
Multifocal	132 (33.2)	232 (39.2)	0.060	237 (36.5)	118 (37.7)	0.722
CLT	113 (28.5)	171 (28.9)	0.946	186 (28.6)	92 (29.4)	0.820
Incidental	127 (31.9)	111 (18.8)	<0.001	166 (25.5)	69 (22.0)	0.231
Surgery			<0.001			<0.001
Lobectomy	67 (16.8)	14 (2.4)		64 (9.8)	10 (3.2)	
Total thyroidectomy	331 (83.2)	578 (97.6)		586 (90.2)	303 (96.8)	
Radioiodine	183 (46.0)	462 (78.0)	<0.001	420 (64.6)	218 (69.6)	0.127
TNM			0.584			0.842
I	387 (97.2)	571 (96.5)		631 (97.1)	303 (96.8)	
II	11 (2.8)	21 (3.5)		19 (2.9)	10 (3.2)	
Hypoparathyroidism	36 (9.0)	40 (6.8)	0.183	47 (7.2)	31 (9.9)	0.166
Health problem	—	—		66 (10.2)	21 (6.7)	0.093
Dose instability*	—	—		118 (19.4)	184 (66.7)	<0.001

CLT, chronic lymphocytic thyroiditis; TNM, tumor–node–metastasis staging system.

* At the last visit, the number of patients with more than 2 years of follow-up was 884 (608 with adequate control and 276 with inadequate control).

### Multivariable logistic regression analysis

The results of the multivariable analysis in patients classified as having an excellent or indeterminate response are presented in Table 5. At 12 months, adequacy was significantly associated with male sex (OR: 2.71 (1.89–3.89); *P* < 0.001). Treatment with total thyroidectomy (0.26 (0.14–0.50); *P* < 0.001) and RAI (0.36 (0.26–0.50); *P* < 0.001) showed a significant but inverse association with adequacy in these patients. Conversely, at the last follow-up visit, adequacy was directly associated with age, although the effect size was modest (1.02 (1.00–1.03); *P* = 0.017), and was inversely and significantly associated with dose instability (0.12 (0.08–0.16); *P* < 0.001). Hypoparathyroidism was not associated with levothyroxine treatment adequacy in either univariate or multivariable analyses.

**Table 5:** Results of univariable and multivariable logistic regression models to study the influence of several covariates as potential predictors of adequacy of levothyroxine therapy in patients who achieved an excellent or indeterminate response at 12 months (A) and at the last visit (B).

	Univariable			Model 1			Model 2		
	OR	95% CI	*P*	OR	95% CI	*P*	OR	95% CI	*P*
(A) At 12 months, *n* = 990									
Gender, male	2.00	1.45–2.75	<0.001	2.00	1.41–2.75	<0.001	2.71	1.89–3.89	<0.001
Age, years	1.01	0.99–1.01	0.316	1.01	0.99–1.01	0.341	1.00	0.99–1.01	0.596
Histology, follicular	0.59	0.37–0.97	0.035				0.77	0.44–1.37	0.377
Tumor size, cm	0.72	0.64–0.81	<0.001				0.84	0.73–0.97	0.018
Multifocal	0.77	0.59–1.01	0.054				0.99	0.73–1.33	0.941
Incidental	2.03	1.51–2.73	<0.001				1.26	0.89–1.79	0.196
Total thyroidectomy	0.12	0.07–0.22	<0.001				0.26	0.14–0.50	<0.001
Radioiodine	0.24	0.18–0.32	<0.001				0.36	0.26–0.50	<0.001
Hypoparathyroidism	1.38	0.86–2.20	0.182				1.41	0.85–2.35	0.182
(B) At the last visit, *n* = 963									
Gender, male	1.14	0.80–1.61	0.471	1.13	0.79–1.60	0.510	1.19	0.77–1.84	0.423
Age, years	1.01	1.00–1.02	0.030	1.02	1.01–1.03	0.002	1.02	1.00–1.03	0.017
Time of follow-up, months	1.00	1.00–1.01	0.001	1.00	1.00–1.01	<0.001	1.00	0.99–1.01	0.174
Histology, follicular	1.36	0.81–2.29	0.243				1.21	0.64–2.29	0.553
Tumor size, cm	0.96	0.86–1.08	0.518				0.92	0.79–1.07	0.296
Multifocal	0.95	0.72–1.25	0.709				1.01	0.71–1.43	0.970
Incidental	1.22	0.89–1.68	0.223				1.00	0.66–1.52	0.995
Total thyroidectomy	0.30	0.15–0.60	0.001				0.47	0.19–1.14	0.096
Radioiodine	0.80	0.60–1.06	0.122				1.05	0.68–1.60	0.842
Hypoparathyroidism	0.71	0.44–1.14	0.156				0.75	0.42–1.34	0.328
Health problems	1.56	0.95–2.65	0.076				1.53	0.82–2.86	0.186
Dose instability*	0.12	0.09–1.17	<0.001				0.12	0.08–0.16	<0.001

OR odds ratio; CI, confidence interval.

Model 1: demographic features (gender and age); Model 2: in addition to the above, clinical, pathological, and therapeutic variables.

* *n* = 884.

## Discussion

The recent ATA guideline revisions have important clinical implications (8). Under the new framework, patients with excellent or indeterminate response will generally receive lower levothyroxine doses, thereby reducing risks associated with iatrogenic thyrotoxicosis (11, 12, 13). Results herein obtained are a clear example of how these conceptual changes translate into real-life clinical practice. The proportion of patients with excellent response increased from 62.3 to 72.0% at 12 months and from 77.8 to 86.9% at the last follow-up (ATA 2015 criteria vs 2025). Treatment adequacy rose from 26.0 and 39.6% to 40.7 and 67.4%, respectively. Under ATA-2015, overtreatment predominated among excellent responders and undertreatment among indeterminate responders (61.8 and 48.4% at 12 months; 30.5 and 62.2% at the last visit). With ATA-2025, overtreatment decreased (51.9 and 25.5%) and undertreatment dropped markedly (6.6 and 8.8%). Levothyroxine dose instability remained the main determinant of long-term inadequacy. Most of the cohort fell into the excellent or indeterminate categories – now both associated with TSH targets within the normal range – representing 97.4% at 12 months and 98.5% at the last follow-up. Only a small subgroup showed biochemical or structural incomplete response, for whom TSH suppression below the reference range is recommended. In this limited group, no meaningful differences were observed between adequately and inadequately treated patients, so our analysis focused primarily on excellent and indeterminate responders.

In the group of patients with excellent or indeterminate response, the above-mentioned marked increase in the proportion of patients classified as adequately treated observed when applying the ATA-2025 criteria – compared with the 2015 framework – may be primarily attributable to three key conceptual changes: the adoption of more liberal TSH targets based on dynamic response to therapy; the recognition that aggressive TSH suppression offers no clinical benefit in low-risk patients free of disease; and the prioritization of an appropriate balance between oncologic benefit and the potential risks associated with overtreatment (8, 9, 11).

The ATA-2015 guidelines recommended maintaining TSH between 0.5 and 2.0 mIU/L in patients with excellent response, whereas the 2025 guidelines permit TSH levels within the normal range for both excellent and indeterminate responders. This modification is supported by evidence showing that TSH suppression does not improve outcomes in low-risk patients with excellent response but significantly increases the risk of atrial fibrillation (14), osteoporosis (15), vertebral fractures (16), and cardiovascular mortality (17). An important finding of our study is that, under the ATA-2015 criteria, some patients with an excellent response and TSH values slightly above the previous target range would have been labeled as undertreated, potentially prompting an unnecessary increase in levothyroxine dosing. The ATA-2025 guidelines, by broadening acceptable TSH values to the full reference range in excellent and indeterminate responders, avoid such dose intensification. This reclassification does not imply any increase in oncologic risk in low-risk patients – whose recurrence probability is already extremely low – but instead aligns LT4 therapy with individualized, risk-adapted management. Moreover, and to an even greater extent, the proportion of undertreated patients within the indeterminate response group declined dramatically to very low levels. These findings are encouraging, as they suggest that, with the implementation of the ATA-2025 criteria, clinicians are less likely to escalate levothyroxine doses unnecessarily in these patients. Beyond biochemical adequacy, broader TSH targets under the ATA-2025 framework may have positive implications for quality of life, as excessive suppression has been associated with symptoms of subclinical hyperthyroidism and reduced well-being, whereas maintaining TSH within the reference range generally provides greater physiological stability and improved patient-reported outcomes (12, 18). Consequently, the implementation of the ATA-2025 criteria enables a safer and more individualized management approach (8, 9, 12, 19).

Interestingly, levothyroxine dose instability remains the principal determinant of treatment adequacy at the last visit among patients with an excellent or indeterminate response. This finding is consistent with our initial analysis using the 2015 criteria (9). We also found that treatment adequacy at 12 months was associated with male sex and, inversely, with RAI and total thyroidectomy. The differences between the predictors of levothyroxine adequacy identified in this study and those reported in our previous publication using the same cohort should be interpreted in light of methodological differences rather than as evidence of new biological determinants. The present analysis evaluated only patients with an excellent or indeterminate response under the broader ATA-2025 framework, whereas our earlier study applied the narrower ATA-2015 criteria to the entire cohort. Consequently, the analytic populations differ, as do the definitions of ‘adequacy’, explaining the discrepancies in statistical associations. These differences reflect the impact of reclassification rather than true changes in the underlying predictive factors.

As expected, a substantial proportion of lobectomy patients did not require levothyroxine and maintained TSH within the reference range. This aligns with published evidence showing that euthyroidism without replacement is common after lobectomy and is associated with improved quality of life (20). The broader TSH targets recommended in the ATA-2025 guidelines further enhance the likelihood of achieving biochemical adequacy in this group, reinforcing the value of individualized management.

Our findings parallel previous reports. In a Spanish cohort, 30.7% of low-risk patients with excellent response were unnecessarily suppressed at the first follow-up, decreasing to 16.3% later (21). In Thailand, only 8.8 and 19.6% of low-risk patients met appropriate TSH targets at the early and late follow-ups (22), and in a Brazilian cohort, only 27% were adequately controlled (23). Other studies also describe high inadequacy rates (11, 24, 25). A recent systematic review highlighted the difficulty of maintaining ATA-2015 TSH targets due to interindividual variability, risks of excessive suppression, and a trend toward less aggressive management in low-risk DTC (26).

We believe that our study may hold practical relevance. Our findings align with the progressive trend toward relaxing TSH suppression in low-risk patients. In a cohort of over 26,000 DTC cases (27), maintaining TSH at 0.5–2 versus 2–4 mIU/mL did not change clinically meaningful recurrence rates in low-risk individuals. Likewise, a recent meta-analysis found no significant effect of TSH suppression intensity on recurrence in low-risk DTC (12), supporting proposals to liberalize target TSH levels after thyroidectomy in this population. Therefore, aligning clinical practice with the updated ATA-2025 criteria reduces the proportion of patients classified as over- or undertreated under previous criteria. This translates into a greater proportion of patients achieving therapeutic targets and a lower risk of associated complications (28).

It is important to underscore that the observed increase in treatment adequacy does not represent a clinical improvement in levothyroxine management per se, but rather a reclassification arising from the broader TSH targets and updated response categories defined by the ATA-2025 guidelines. Our study was designed precisely to quantify this reclassification effect in a real-world cohort. Although classification-driven, these changes have practical implications: fewer patients will be labeled as inadequately treated, reducing unnecessary dose modifications and aligning clinical practice with a more individualized, risk-adapted therapeutic approach.

This study has notable strengths, including a large multicenter cohort of 1,016 real-world patients, long follow-up, clearly defined inclusion criteria, and rigorous data collection by thyroid specialists. Its principal contribution is the direct comparison of levothyroxine adequacy under the ATA-2015 and ATA-2025 frameworks, illustrating how updated guideline principles may reduce the proportion of patients with both over- and undertreatment in low-risk DTC.

However, several limitations apply. As a retrospective observational design, it cannot support causal inference, and inter-center variability in assays, titration practices, and follow-up schedules may have influenced adequacy estimates. The absence of standardized LT4 adjustment protocols and the small number of patients with incomplete responses further limit extrapolation. This study evaluates the impact of the ATA-2025 criteria on the classification of levothyroxine adequacy but does not assess clinically meaningful outcomes, such as recurrence, cardiovascular events, bone health, or quality of life. Therefore, no inference can be made regarding whether the broader TSH targets improve, worsen, or have a neutral effect on long-term outcomes. The observed changes reflect reclassification rather than demonstrated clinical benefit. Future prospective studies are needed to determine how these guideline updates influence oncologic safety, metabolic complications, and patient-reported outcomes.

Although the ATA-2025 guidelines refine the initial recurrence risk categories – introducing histology-specific distinctions that may reclassify a small subset of papillary or follicular tumors into low-intermediate or intermediate-high risk – we deliberately retained the ATA-2015 low-risk definition for cohort selection. This choice ensures consistency with our previous study and reflects the primary aim of the present analysis, which was to examine the impact of the updated 2025 DRS and TSH target recommendations. As these elements represent the major conceptual changes in the new guideline, the limited reclassification of a few tumors at baseline does not materially affect the applicability or interpretation of our findings. Future studies may explore how ATA-2025 reclassifies baseline risk, but this was beyond the scope of the present work.

In summary, our findings reflect a real-world, proactive shift in levothyroxine management that preceded formal publication of the ATA-2025 guidelines. Although most patients in our cohort were treated under earlier guidelines, treatment patterns were already gradually moving toward less stringent TSH suppression in patients with excellent or indeterminate response. The ATA-2025 criteria formalize and reinforce this evolution by broadening TSH targets in favorable responders and prioritizing individualized, risk-adapted management. The marked increment in the proportion of patients classified as adequately controlled observed when applying the 2025 framework therefore mirrors changes already taking place in routine practice and suggests that further alignment with the updated guidelines will continue to reduce both overtreatment and undertreatment in low-risk DTC. Overall, our results suggest that many low-risk patients can now be managed with greater therapeutic flexibility and better quality of life, reflecting a more balanced and evidence-based approach.

## Supplementary materials



## Declaration of interest

The authors declare that this research was conducted in the absence of commercial or economic relationships that could generate a conflict of interest.

## Funding

The present investigation has not received any financial support from public sector agencies, commercial sector, or non-profit entities.

## Author contribution statement

JJD and JS conceptualized the study. JJD performed data analysis and drafted the manuscript. All authors participated in data collection and provision of study materials, critically reviewed the manuscript, and approved the final draft.

## Data availability

Data are available through a reasonable request to the corresponding author.
