# Supplementary material for: Improved adequacy of levothyroxine treatment in low-risk differentiated thyroid cancer using the 2025 ATA criteria: a multicenter real-world analysis of 1,016 patients
Source: Eur Thyroid J. 2026 Jun 30;15(3):ETJ260066. doi: 10.1530/ETJ-26-0066 (PMC13326724; doi:10.1530/ETJ-26-0066)
Supplement: Supplementary file 1 [file supplementary_materials.pdf]

Improved Adequacy of Levothyroxine Treatment in Low-Risk Differentiated Thyroid Cancer Using the 2025 ATA Criteria: A Multicenter Real-World Analysis of 1,016 Patients

SUPPLEMENTARY MATERIAL

Table S1. Methods for measuring serum thyrotropin by the investigators participating in the study, indicating the reference interval of the assay used

**Manufacturer**	**Instrument**	**Investigator (number, %)**	**Patients (number, %)**	**Reference interval (mcU/ml)**
Abbott	Alinity	3 (17.6)	155 (15.3)	0.35-4.94
Beckman	DXI	3 (17.6)	257 (25.3)	0.30-5.30
Roche	Cobas	2 (11.8)	86 (8.5)	0.25-5.00
	Elecsys	2 (11.8)	153 (15.1)	0.3-4.2
Siemens	Advia Centaur	1 (5.9)	61 (6.0)	0.55-5.0
	Atellica	6 (35.3)	304 (29.9)	0.55-4.78

Table S2. Comparison of patients with adequacy and inadequacy of levothyroxine treatment in the group of subjects with biochemical or structural incomplete response at 12 months and at the last follow-up visit according to the ATA 2025 criteria (TSH below normal reference range)

	Adequacy of levothyroxine therapy in patients with biochemical or strtuctural incomplete response					
	Evaluation at 12 months (n=26)			Evaluation at last visit (n=15)		
	Adequate (n=16)	Inadequate (n=10)	P value	Adequate (n=9)	Inadequate (n=6)	P value
**Gender**			1.0			0.486
**Male**	13 (81.3)	8 (80.0)		2 (22.2)	0	
**Female**	3 (18.8)	2 (20.0)		7 (77.8)	6 (100)	
**Age, yr**	46.0 (30.3-54.3)	49.0 (28.8-53.3)	0.816	42.0 (33.5-56.5)	46.5 (29.3-60.3)	0.864
**Time of follow-up, mo**	—	—		56.5 (4.0-107.5)	82.5 (41.3-238.8)	0.689
**Histology**			0.508			1.0
**Papillary**	14 (87.5)	10 (100)		7 (77.8)	4 (66.7)	
**Follicular**	2 (12.5)	0		2 (22.2)	2 (33.3)	
**Tumor size, cm**	1.5 (0.73-2.43)	1.9 (0.95-3.28)	0.452	2.0 (0.5-4.0)	1.9 (0.6-4.0)	0.955
**Multifocal**	4 (25.0)	4 (40.0)	0.664	4 (44.4)	1 (16.7)	0.580
**CLT**	5 (31.3)	2 (20.0)	0.668	3 (33.3)	1 (16.7)	0.640
**Incidental**	3 (18.8)	1 (10.0)	1.0	1 (11.1)	2 (33.3)	0.525
**Surgery**						
**Lobectomy**	0	0		0	0	
**Total thyroidectomy**	16 (100)	10 (100)		9 (100)	6 (100)	
**Radioiodine**	14 (87.5)	8 (80.0)	0.625	7 (77.8)	3 (50.0)	0.329
**TNM**						
**I**	16 (100)	10 (100)		9 (100)	6 (100)	
**II**	0	0		0	0	
**Hypoparathyroidism**	2 (12.5)	5 (50.0)	0.069	2 (22.2)	0	0.486

**Health problem**	—	—		0	1 (16.7)	0.357
**Dose instability***	—	—		3 (33.3)	5 (83.3)	0.138

Data are the median (IQR) for quantitative variables, and the number (percentage) for categorical variables.
Abbreviations: CLT, chronic lymphocytic thyroiditis; TNM, tumor-node-metastasis staging system.
*At the last visit, the number of patients with more than 2 years of follow-up was 14 (8 with adequate control and 6 with inadequate control).
